# Internal jugular vein reconstruction using a triple-paneled great saphenous vein graft

**DOI:** 10.1186/s12957-023-02902-4

**Published:** 2023-01-16

**Authors:** Shimpei Miyamoto, Takeaki Hidaka, Osamu Fukuoka, Kou Fujisawa, Mutusmi Okazaki

**Affiliations:** 1 Department of Plastic and Reconstructive Surgery, Graduate School of Medicine, The University of Tokyo, 7-3-1 Hongo, Bunkyo-ku, Tokyo, 113-8655 Japan; 2 Department of Otolaryngology - Head and Neck Surgery, Graduate School of Medicine, University of Tokyo, Tokyo, Japan

**Keywords:** Internal jugular vein, Great saphenous vein, Vein graft, Reconstruction, Size discrepancy, Paneled method

## Abstract

**Background:**

Donor–recipient diameter discrepancy can be problematic when using an autologous great saphenous vein graft for internal jugular vein reconstruction. A triple-paneled method of saphenous vein grafting is one solution.

**Case presentation:**

A 54-year-old man with a thyroid papillary carcinoma underwent total thyroidectomy and bilateral neck dissection. An 8-cm segment of the right internal jugular vein was resected. For reconstruction, a 30-cm segment of the great saphenous vein was harvested and divided into three pieces of equal length. After opening each piece longitudinally, they were sutured together in a side-by-side fashion to create a cylinder that was used to reconstruct the internal jugular vein defect. The graft was patent 10 months after the surgery.

**Conclusion:**

The triple-paneled method is feasible for autologous great saphenous vein graft reconstruction of the internal jugular vein.

**Supplementary Information:**

The online version contains supplementary material available at 10.1186/s12957-023-02902-4.

## Background

Simultaneous resection of both internal jugular veins (IJVs) can cause acute reduction in cerebral venous drainage and result in blindness, inappropriate anti-diuretic hormone hypersecretion, cerebral edema, cerebral vessel complications, and even death. Potential non-cerebral complications include facial and laryngeal edema [1]. Therefore, when bilateral IJV resection is performed, at least one IJV should be reconstructed.

Although various methods have been reported for IJV reconstruction, autologous vein grafting is ideal. The most common graft used is the great saphenous vein (GSV). However, matching its diameter with that of the IJV can be problematic [2–4]. We describe a triple-paneled method of saphenous vein grafting that addresses this issue.

## Case presentation

**Fig. 1:**
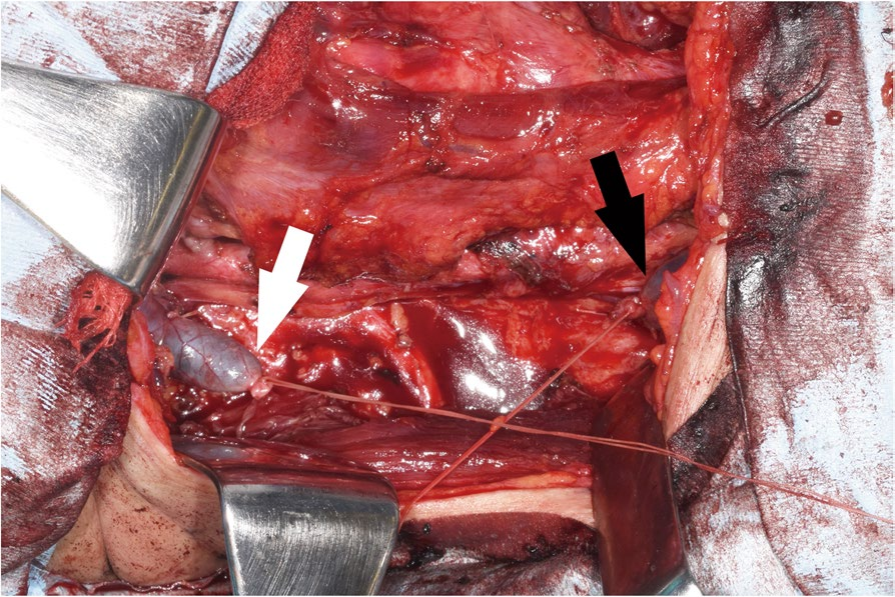
Intraoperative photograph after tumor resection. The left side is cephalad. The black and white arrows indicate the proximal and distal stumps of the right internal jugular vein, respectively

A 54-year-old man with a thyroid papillary carcinoma underwent total thyroidectomy and bilateral neck dissection. Both IJVs were invaded by metastatic cervical lymph nodes. The right IJV was completely resected while the left one only required partial resection. The resulting right IJV defect spanned approximately 8 cm (Fig. 1).

**Fig. 2:**
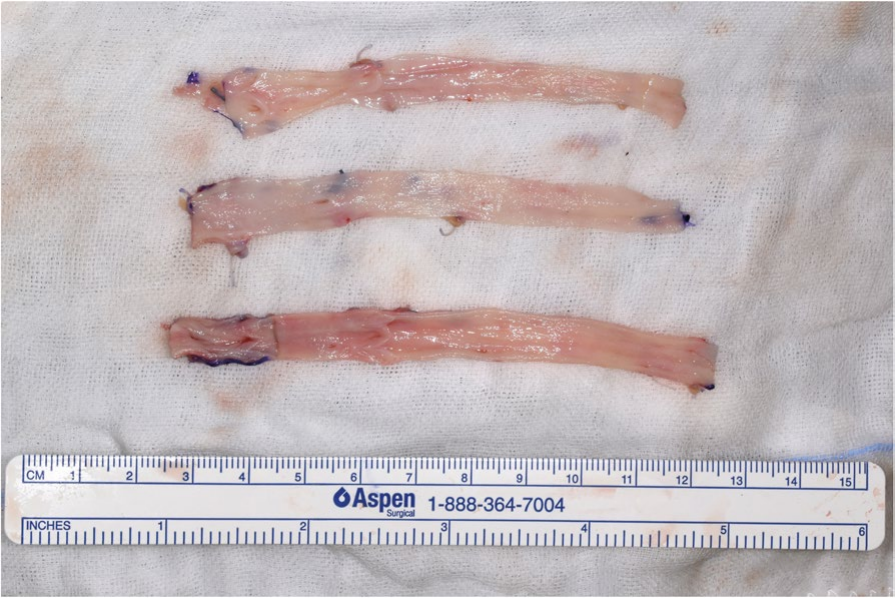
After harvesting a 30-cm section of the great saphenous vein, it was divided into three pieces of equal length. Each piece was then opened longitudinally

**Fig. 3:**
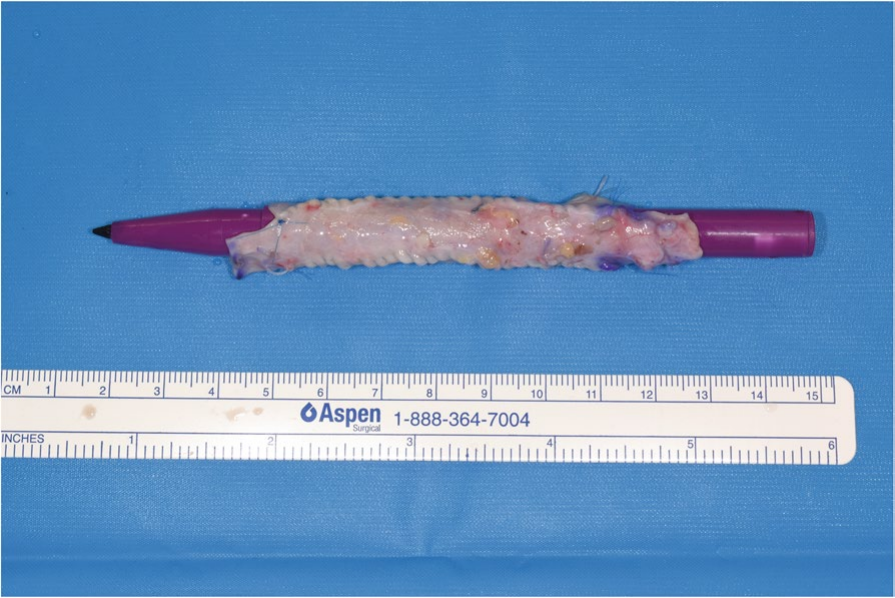
The triple-paneled graft was created by suturing the three graft pieces together into a cylinder

**Fig. 4:**
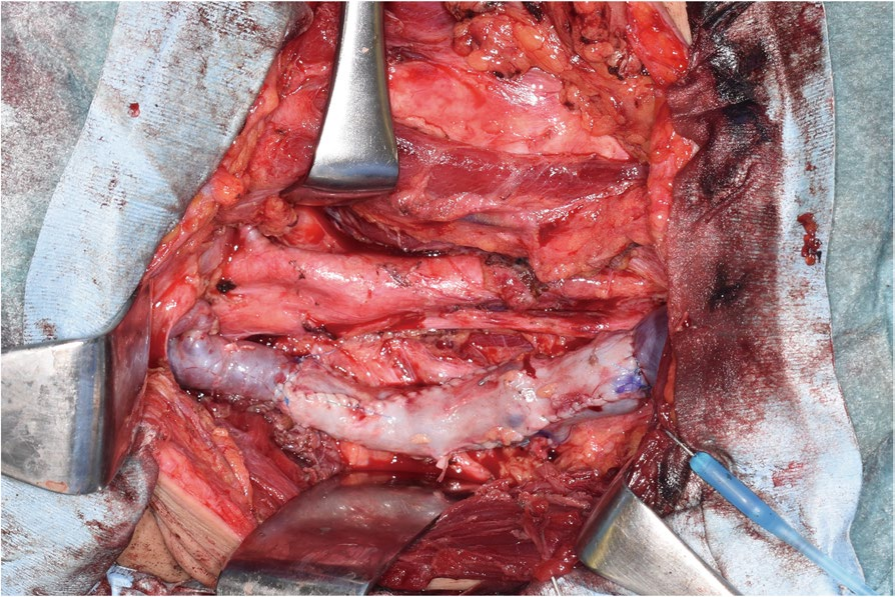
Intraoperative photograph after reconstruction of the right internal jugular vein. The left side is cephalad

The defect was immediately reconstructed using a 30-cm section of the right GSV, which was harvested through three small incisions in the medial thigh. The graft was then divided into three pieces of equal length. After opening each piece longitudinally, they were sutured together in a side-by-side fashion using continuous 6-0 polypropylene suture to create a single cylinder, finally wrapping it around a surgical marker pen (Figs. 2 and 3). The triple-paneled graft was then interposed to connect the proximal and distal stumps of the right IJV. Each end of the graft was anastomosed to the stumps in an end-to-end fashion using continuous 6-0 polypropylene suture (Fig. 4). The wall defect of the left IJV was repaired using an autologous patch graft taken from the ipsilateral external jugular vein. No anticoagulation or antiplatelet therapy was administered during surgery or after.

The patient’s postoperative course was uneventful. IJV patency was monitored using color Doppler ultrasonography (Video 1). Ten months after surgery, both IJVs were patent and the right IJV diameter was 15 mm; he reported no symptoms of increased intracranial pressure.

## Discussion

The GSV is the most frequently used donor vein for IJV reconstruction. Although such a reconstruction can be performed with an intact GSV [5, 6], this is not ideal because the diameter of the bypass conduit is much smaller than that of the native IJV, which reduces flow and threatens long-term patency [7]. Therefore, techniques have been devised to deal with the size discrepancy.

The most frequently used technique for reconstructing large veins using small vein grafts is the spiral method [2, 7], which entails longitudinal opening of the harvested GSV graft, wrapping it in a spiral around a temporary tubular stent, and constructing a tube graft. IJV reconstruction using this technique has been reported by several authors [2, 4]. The spiral method enables tailoring the graft diameter to the required size; however, the sutures are exposed to the bloodstream and the suture lines can be quite long. Moreover, reconstruction of a long IJV defect can be particularly cumbersome. Another disadvantage is that it is difficult to know the length of the graft needed.

The paneled method is another commonly used technique [3, 8]. With this method, the graft is opened longitudinally and divided into several pieces. The pieces are then sutured together side by side to create a cylinder of larger diameter. Urayama et al. described a double-paneled method to reconstruct an IJV in a patient who underwent bilateral radical neck dissection for tongue cancer [3]. They divided the GSV graft into two equal parts and sutured them together to double the diameter. The main advantage of the double-paneled method is its simplicity. In addition, the suture lines are shorter than those of the spiral method. The length of each piece of the graft should be the same as the length of the defect, which can be easily determined. The main disadvantage is that the maximum graft diameter is only twice that of the GSV, which is still usually narrower than that of the IJV.

In our patient, the right IJV was reconstructed using a triple-paneled method, which perfectly fit the required diameter. Pantoja et al. reported the use of triple-paneled saphenous vein grafts to reconstruct the portomesenteric venous system after pancreaticoduodenectomy [8]. To the best of our knowledge, this is the first report of a triple-paneled GSV graft being used for IJV reconstruction. The average diameter of the right IJV is approximately 17 mm, while that of the GSV 5 cm distal to the saphenofemoral junction is approximately 5 mm [9, 10]. Therefore, the triple-paneled GSV method is better suited for IJV reconstruction than the double-paneled method.

The main drawback of the triple-paneled method is that the graft needs to be at least three times as long as the defect. A GSV graft approximately 35 cm in length can be harvested using three or four small incisions between the inguinal area and the knee. If additional incisions are made in the lower leg, a 70-cm graft can be harvested, which should be adequate for most IJV defects.

## Conclusions

Triple-paneled GSV grafts are simple to construct and feasible for use in IJV reconstruction. Furthermore, they have potential to expand the use of autologous vein grafts in the reconstruction of other large vessels. Future studies are warranted to examine their long-term patency rate.

## Supplementary Information

**Additional file 1: Video 1.** IJV patency was confirmed by color Doppler ultrasonography at 10 months postoperatively.

## Data Availability

Further case information is available from the corresponding author on reasonable request.

## Abbreviations

IJV: Internal jugular vein
GSV: Great saphenous vein
